# A millennium of cold-water coral habitat loss in the East Pacific during low ENSO variability in the mid- to late Holocene

**DOI:** 10.1073/pnas.2532081123

**Published:** 2026-04-20

**Authors:** Joseph A. Stewart, Laura F. Robinson, Michelle L. Taylor, Daniel J. Fornari, Katleen Robert, Stuart Banks, Tianyu Chen, Tao Li, James Kershaw, Shannon Hoy, Qian Liu, Jessica D. Gordon, Maria Luiza de Carvalho Ferreira, Ana Samperiz, Yingchu Shen, Yun-Ju Sun, Maoyu Wang

**Affiliations:** ^a^School of Earth Sciences, University of Bristol, Bristol BS8 1RJ, United Kingdom; ^b^Department of Environment and Geography, University of York, York YO10 5NG, United Kingdom; ^c^School of Life Sciences, University of Essex, Colchester CO4 3SQ, United Kingdom; ^d^Department of Geology and Geophysics, Woods Hole Oceanographic Institution, Woods Hole, MA 02543; ^e^Fisheries and Marine Institute of Memorial University, St. John’s, NL A1C 5R3, Canada; ^f^Charles Darwin Foundation, Puerto Ayora, Galápagos, Ecuador; ^g^School of Earth Sciences and Engineering, Nanjing University, Nanjing 210023, China; ^h^State Key Laboratory of Palaeobiology and Stratigraphy, Nanjing Institute of Geology and Palaeontology, Nanjing 210008, China; ^i^Seirios Solutions, Brookfield, NH 03872; ^j^Department of the Geophysical Sciences, University of Chicago, Chicago, IL 60637; ^k^Department of Earth, Ocean and Atmospheric Science, Florida State University, Tallahassee, FL 32310; ^l^School of Earth and Environmental Sciences, Cardiff University, Cardiff CF10 3AT, United Kingdom; ^m^Earth and Environmental Science, University of St. Andrews, St. Andrews KY16 9TS, United Kingdom

**Keywords:** cold-water corals, El Niño–Southern Oscillation, U-Th dating, Galápagos

## Abstract

The evolution of El Niño–Southern Oscillation (ENSO) under past and future climate change and its influence on marine ecosystems is highly debated. To examine links between tropical climate variability, high-latitude feedbacks, and deep-sea ecosystems, we have analyzed over 900 subfossil cold-water scleractinian corals from the Galápagos Archipelago. U-Th dating shows that these corals have inhabited the region for at least 117,000 y, thriving at depths down to ~1,000 m. However, we find a millennial-scale gap in coral occurrence ~5,000 y ago, coinciding with reduced ENSO variability and a persistent La Niña-like state. Coral presence resumed as ENSO cyclicity strengthened again toward modern conditions. While shallow-water coral growth has been shown to generally decline in the equatorial Pacific during El Niño surface warming, the mid- to late Holocene loss of cold-water corals may reflect the opposite: prolonged La Niña-like conditions. We hypothesize that stronger upwelling at this time intensified the oxygen minimum zone, adversely affecting cold-water coral habitats. With ENSO projected to change under continued ocean warming, the potential vulnerability of cold-water corals to future climate change is a growing concern. This study underscores the value of understanding past ENSO behavior and its impact on deep-sea ecosystems in a warming world.

## Significance

Cold-water corals are ecosystem engineers that currently thrive in the East Equatorial Pacific, yet their history and climate sensitivity remain poorly understood. By compiling over 900 coral ages from the Galápagos region, we document continuous coral presence since ~117 thousand years ago (ka) but reveal a striking mid- to late Holocene (~5 to 3.5 ka) hiatus. This gap in coral habitat coincides with suppressed El Niño–Southern Oscillation (ENSO) variability, intensified La Niña–like conditions, and reduce dissolved oxygen in seawater, highlighting the vulnerability of these ecosystems to tropical climate dynamics. Our results provide rare insights into the coupled impacts of ENSO upwelling and oxygen depletion on deep-sea ecosystems in the iconic Galápagos region.

Cold-water corals are critical components of marine ecosystems, forming complex structures that provide habitat and support biodiversity in some of the ocean’s most remote and least studied regions (1, 2). Despite their importance to the deep sea, most coral research has focused on the more accessible shallow-water reefs, thus our understanding of cold-water coral ecosystems and their response to climate perturbations remains limited.

Cold-water coral distribution is shaped by environmental factors such as temperature, depth, salinity, substrate, slope, oxygen, and carbonate chemistry (3). Global habitat models highlight aragonite saturation state and temperature as primary controls (4), while regional studies also identify slope, salinity, oxygen, and depth (5–7). Broader species distribution approaches further emphasize carbonate saturation state, surface water primary production (food supply), and terrain ruggedness (8, 9), though investigation has been strongly biased toward the North Atlantic, with few studies in the Pacific (10). Experimental work has revealed complex physiological responses, for instance, colonial *Desmophyllum pertusum* (formerly *Lophelia pertusa*) can calcify under elevated CO_2_ and warming conditions, but only if sufficient food is available (11). The aerobic function of this coral also notably declines below ~140 μmol kg^−1^ O_2_ (12). Solitary corals such as *Desmophyllum dianthus* are estimated to live for around 190 y, with new corals recruiting infrequently [every ~30 y (13)], implying recovery after disturbance can take decades. Despite some progress (e.g., refs. 7, 8, 14, and 15), temporal changes in cold-water coral ecosystems, especially in the Pacific Ocean, remain poorly constrained. This severely limits our understanding of habitat loss and recovery rates and thus their resilience to changing climate.

**Fig. 1.**
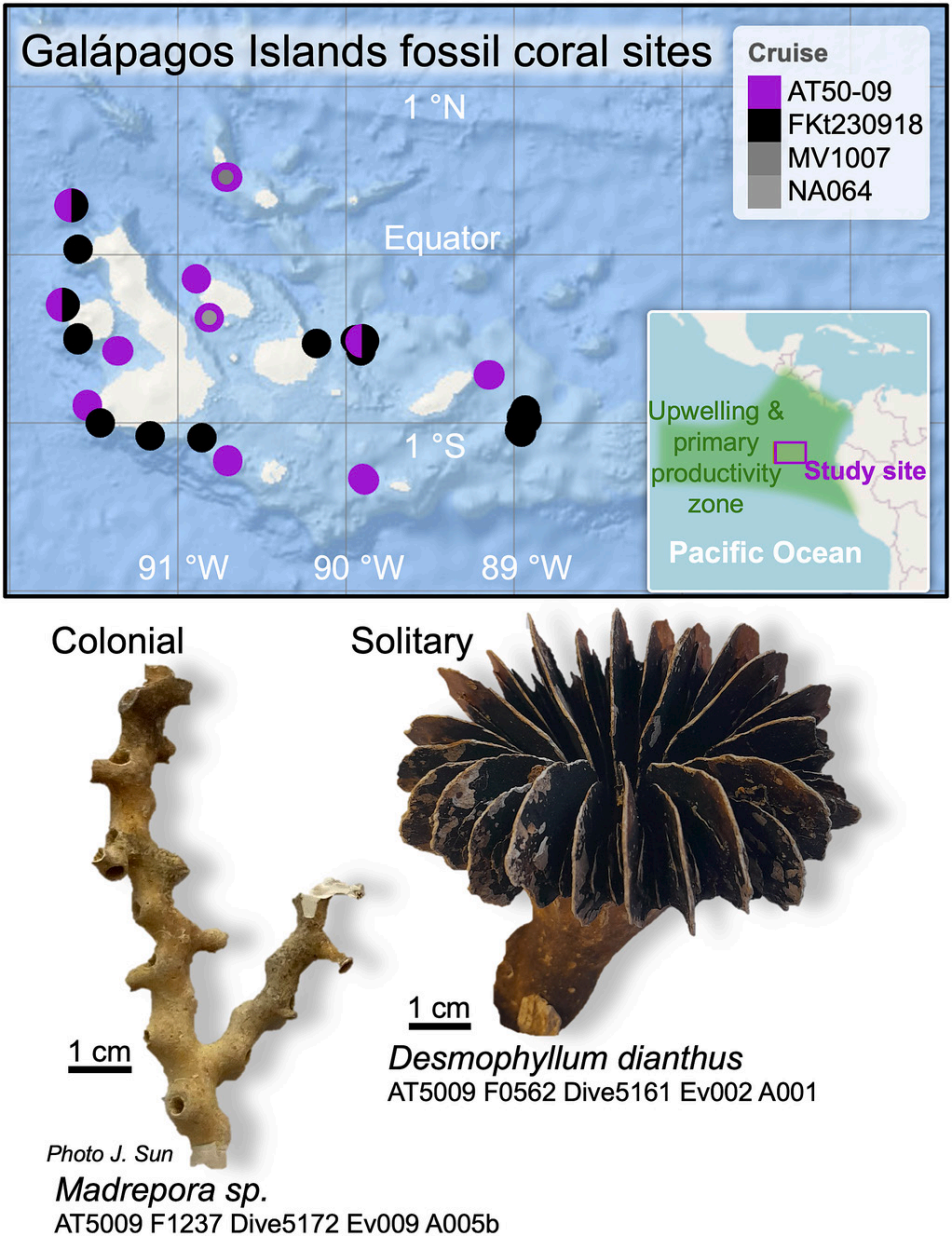
Galápagos Islands study site in the East Equatorial Pacific, scleractinian coral sample locations, and example photographs of colonial and solitary corals used in this study. Base map from ArcGIS Ocean Basemap.

**Fig. 2.**
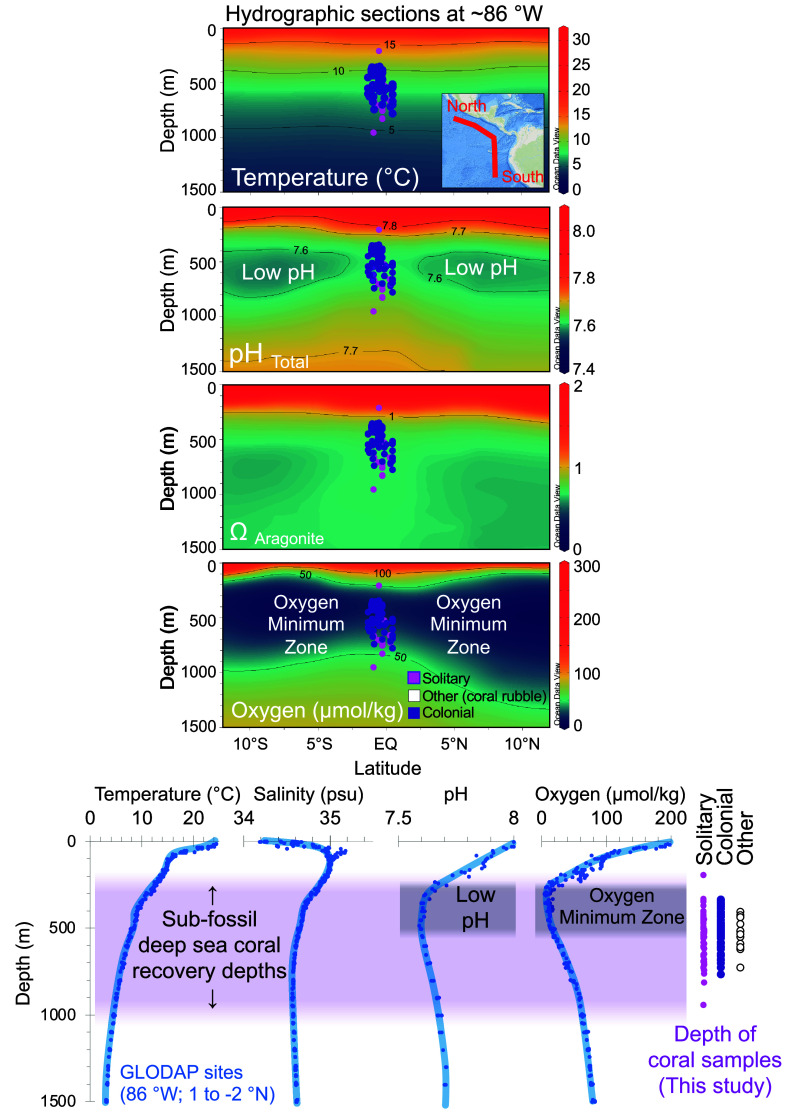
Modern hydrographic context of coral sample locations around the Galápagos Islands (East Equatorial Pacific). North–South hydrographic sections (*Upper* panels; see map insert in *Top* panel) and station data (*Lower*) plotted using seawater bottle data from GLODAPv2.2020 (16) and Ocean Data View version 5.6.7.

**Fig. 3.**
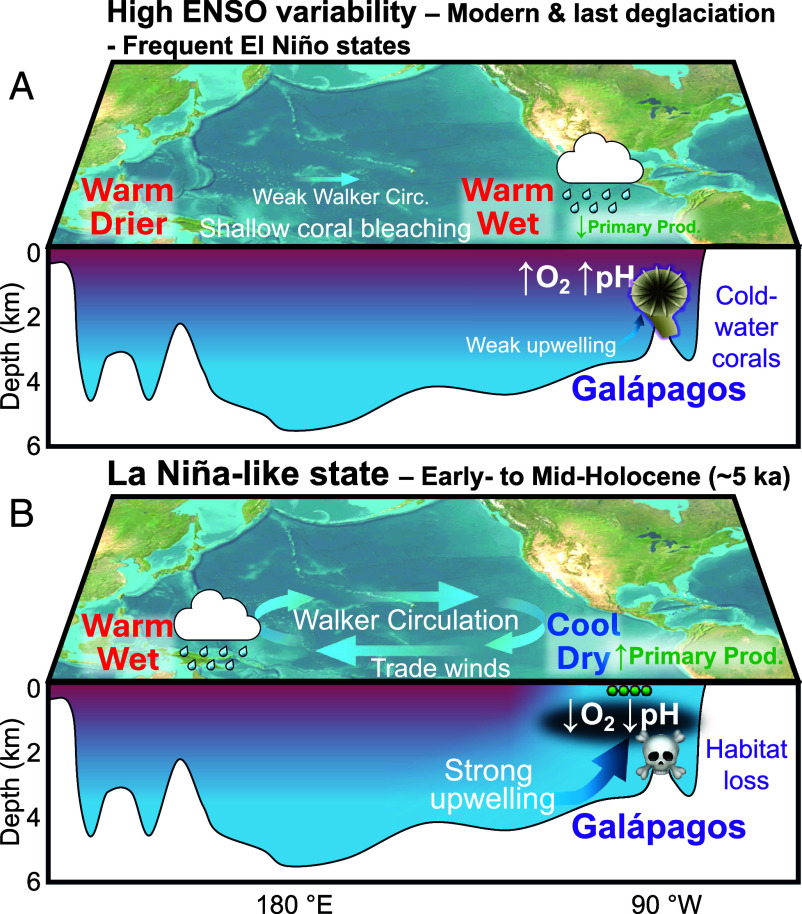
Schematic ocean cross-section of (*A*) High ENSO variability during the last deglaciation and (*B*) Low ENSO variability permanent La Niña-like state and its impact on East Equatorial Pacific cold-water corals. (*A*) During high ENSO variability intervals El Niño events are common, decreasing east-west sea surface temperature gradients, increasing rainfall in the east, and decreasing upwelling in the East Pacific. (*B*) During a permanent La Niña-like state Walker Circulation is strong, east-west sea surface temperature gradients are large, the East Pacific is cool and dry, enhanced upwelling drives high primary production and intensifies the oxygen minimum zone in the East Equatorial Pacific. Satellite imagery basemap from ArcGIS.

The Galápagos Archipelago (Fig. 1) represents a unique and ecologically important location for studying cold-water coral ecosystems, where deep waters, rich in carbon and nutrients, reconnect with the surface and atmosphere, thus influencing both marine conditions and global climate. This region in the East Equatorial Pacific has an intense modern oxygen minimum zone (down to values below 20 μmol kg^−1^) and low pH (down to 7.6 and undersaturation with respect to aragonite) at 250 to 500 m depth due to organic matter remineralization [Fig. 2; (16, 17)]. It is also strongly influenced by ENSO, which drives large fluctuations in temperature and upwelling, and consequently changes in primary production and dissolved oxygen (17) (Fig. 3). A comprehensive temporal record of Galápagos cold-water corals would provide rare insights into how long-term climate variability and environmental stressors shape ecosystem resilience.

Here, we present radiometric dates of subfossil cold-water coral skeletons collected from deep waters around the Galápagos Islands and compare them with ENSO paleoclimate records spanning the last deglaciation to evaluate the timing and drivers of coral abundance.

## Results

**Fig. 4.**
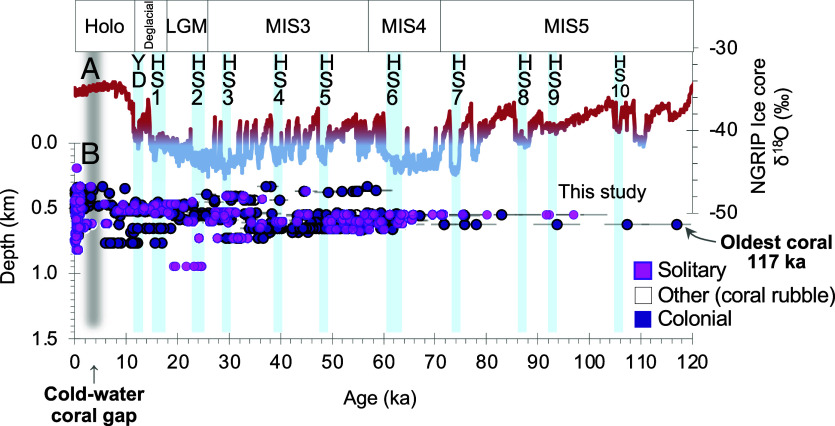
East Equatorial Pacific scleractinian cold-water coral occurrence over the last 120 ky (*A*) Greenland ice core δ^18^O temperature (and ice volume) record (19) highlighting HS and the Younger Dryas as vertical blue bars. (*B*) Distribution of Galápagos cold-water coral U-Th ages in this study with sample depth. Coral samples are separated by solitary and colonial. Error bars represent the 2 SD age uncertainty.

We present 910 new U-Th dates [plus 28 previously published (18)] of cold-water scleractinian corals from the Galápagos platform and deeper flanks of the islands, spanning 190 to 950 m depth. The oldest sample, a colonial *Dendrophyllia*, was found to be ~117,000 y old (±6,800 y; AT5009_F0228_Dive5158_Ev005_A001c), indicating that cold-water corals have been present in this region since at least Marine Isotope Stage 5 (Fig. 4).

The age distribution of the corals reveals that a significant portion of the samples (n = 180, or over 30%) were less than 1,000 y old, consistent with the expected preservation bias toward younger samples as older corals become increasingly degraded over time.

**Fig. 5.**
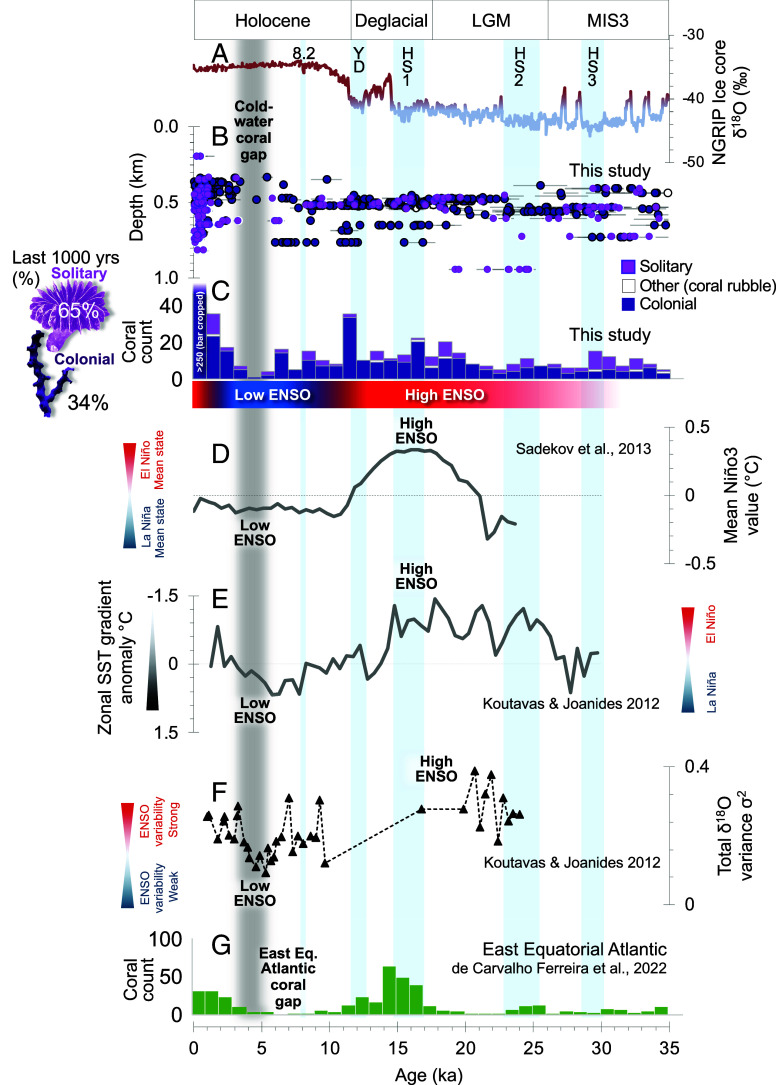
East Equatorial Pacific scleractinian cold-water coral occurrence over the last 35 ky. (*A*) Greenland ice core δ^18^O temperature (and ice volume) record (19) highlighting HS, the Younger Dryas and the 8.2 ky event (8.2) as vertical blue bars. (*B*) Distribution of Galápagos cold-water coral U-Th ages in this study with sample depth and (*C*) 1,000-y binned histogram. Coral samples are separated by solitary and colonial. Error bars represent the 2 SD age uncertainty. (*D*) Mean Niño3 measure of east-west Pacific sea surface temperature gradient using planktic foraminiferal geochemistry (22). (*E* and *F*) Sea surface temperature gradient anomaly in the tropical Pacific and variance estimated using planktic foraminiferal geochemistry (23). (*G*) Histogram of cold-water coral occurrence in the East Equatorial Atlantic (14, 24).

**Fig. 6.**
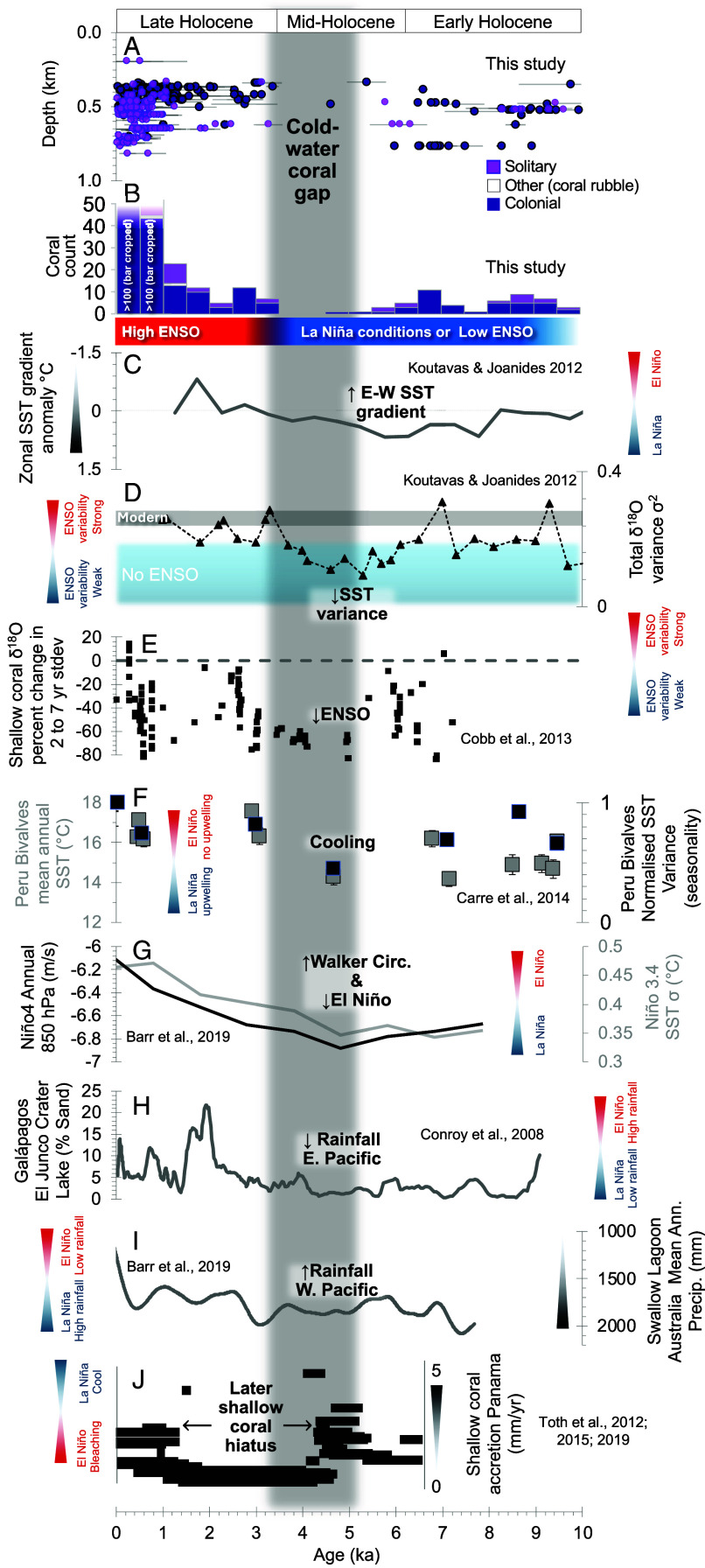
East Equatorial Pacific scleractinian cold-water coral occurrence over the Holocene compared to ENSO records. (*A* and *B*) Distribution of Galápagos cold-water coral U-Th ages as in Fig. 5 (note histogram binned at 500-y intervals). (*C* and *D*) Sea surface temperature gradient anomaly and variance as in Fig. 5 (23). (*E*) Shallow coral δ^18^O temperature variability in the Equatorial Pacific (25). (*F*) δ^18^O sea surface temperature records in fossil bivalve shells from Peru (26). (*G*) CSIRO Mk3L climate system model outputs of Walker Circulation (zonal wind strength) and Niño 3.4 sea surface temperature variance (27). (*H*) Rainfall proxy—percentage sand size fraction in sediments cores from El Junco Crater Lake in the Galápagos (28). (*I*) Precipitation record from leaf carbon isotopes preserved in lake sediments from eastern Australia (27). (*J*) East Equatorial Pacific (Panama) shallow water coral accretion rates (29, 30). Blue and red hourglasses on each panel highlight the sensitivity of each proxy record toward El Niño conditions or high ENO variability (red) and prevailing La Niña conditions or low ENSO variability (blue).

A striking result from the data is the absence of corals between ~5.0 and 3.5 ka (Figs. 5 *B* and *C* and 6 *A* and *B*). During this ~1.5 ky gap, only one colonial coral sample was recovered (FKt230918.579.6.1b), despite abundant occurrences both before (next oldest coral 5,368 ± 415 ka) and after (next youngest coral 3,346 ± 136 ka). This single specimen came from a central site that today hosts a thriving reef at a mid- to shallow depths (~480 m) compared to other dive sites in this study (20, 21).

Both solitary and colonial taxa are represented here, with limited temporal or depth-related habitat differences, save for perhaps an absence of solitary corals below ~500 m during the last deglaciation to early Holocene (Figs. 5*B* and 6*A*). We also examined spatial patterns by separating coral dates from sites north and south of 0.5°S, as well as east (within the archipelago) and west (open-ocean side) of 91°W, but found no evidence of spatial bias over time (*SI Appendix*, Fig. S1). Further partitioning by dive number shows that corals’ age distributions are not a sampling artifact (e.g., if each dive site had only yielded corals of similar age) (*SI Appendix*, Fig. S2). For instance, several AT50-09 Alvin dives (5158, 5159, 5161, 5173) recovered corals immediately before and after, but not during, the mid- to late Holocene coral gap (*SI Appendix*, Fig. S2). This strongly suggests that the hiatus at ~5 to 3.5 ka is not the result of taxonomic, spatial, preservation, or sampling biases, but instead reflects a genuine change in cold-water coral occurrence across the region.

Following the approach of Stewart et al. (7), we adopt a statistical approach to assess the likelihood that the gap in coral occurrence we find at ~5 to 3.5 ka is merely an artifact of sampling (Fig. 5 *B* and *C*). Discounting corals younger than 1,000 y in age that will be strongly favored by preservation bias (n = 289), 364 corals were recovered from deep waters with ages between 30 and 1 ka. By running Monte Carlo simulations (10,000), we estimate the probability that a random distribution of these corals would yield 1 or fewer corals within any 1,000-y interval between 30 and 1 ka to be 0.09%. This means that, under the assumption that corals are randomly and uniformly distributed across this interval, there is a less than 1 in 1,000 chance of seeing 1 or fewer corals in any 1,000-y bin—including the one observed between ~5 and 3.5 ka.

## Discussion

### History of Cold-Water Coral Occurrence in the Galápagos.

Subfossil cold-water corals were recovered from 190 to 950 m, thus spanning diverse deep-sea habitats and crossing the modern oxygen minimum and low-pH zones (~300 m; Fig. 2). Our finding that deep-water corals have continuously inhabited the Galápagos region for at least the past 117,000 y underscores the exceptional longevity and ecological importance of this ecosystem (Fig. 4). These ancient coral communities support rich benthic ecosystems—fish, crustaceans, sponges, and other invertebrates—while enhancing deep-ocean carbon storage through retention and transformation of sinking organic matter (1, 2).

As demonstrated above, the most striking feature of our record is a pronounced ~1.5 ky gap in coral occurrence between ~5.0 and 3.5 ka, after which corals reappeared and persist to the present day. Below we review published records of ENSO variability that show this mid- to late Holocene coral hiatus coincides with reduced ENSO variability and a more persistent La Niña–like state in the Equatorial Pacific (Figs. 5 and 6). This interruption stands in marked contrast to the last three Heinrich events, the Younger Dryas (~12 ka; where we document a notable acme in occurrence of more than 30 corals), the Last Glacial Maximum, and the 8.2 ka event, during which cold-water coral abundance at our site remained high despite pronounced global climate perturbations expressed in ice core and ocean circulation records (19, 31, 32).

### ENSO Variability Records Over the Last Deglaciation and Holocene.

Proxy and modeling evidence show enhanced ENSO variability during the last deglaciation and frequent El Niño events (Fig. 3*A*), particularly during periods of Northern Hemisphere cooling and weakened Atlantic Meridional Overturning Circulation such as Heinrich Stadial 1 (HS1) and, to a lesser extent, the Younger Dryas (YD) (31) (Fig. 5). Reconstructions of east–west tropical Pacific sea surface temperature gradients and foraminiferal δ^18^O variability indicate warming of the East Pacific and an ENSO variability maximum around ~15 ka, followed by a decline into the early to mid-Holocene [Figs. 5 *D*–*F* and 6 *C* and *D* and (22, 23)]. Model simulations support the link between Atlantic overturning slowdown and stronger ENSO showing that North Atlantic cooling is linked to warmer, more stratified, tropical surface and intermediate waters and an El Niño–like mean state in the Pacific (32–34). Shallow-water coral and mollusk-based geochemical temperature records also confirm HS1 as a period of heightened ENSO variability (35, 36).

By contrast, the early to mid-Holocene (~8 to 4 ka) was marked by suppressed ENSO variability, with a distinct minimum around 5 ka (37), and a persistent La Niña–like state in the tropical Pacific (Figs. 3*B* and 6). Central Pacific shallow-water coral δ^18^O shows minimal ENSO-band variance [2 to 7-y; Fig. 6*E*; (25)], while fossil bivalve δ^18^O records off Peru record both lower annual sea surface temperatures and reduced temperature variance, consistent with infrequent El Niño events [Fig. 6*F*; (26)]. This pattern of maximum east–west sea surface temperature gradients and overall lower temperatures in the East Equatorial Pacific in the mid-Holocene is supported by compiled planktic foraminiferal Mg/Ca and δ^18^O records from sediment cores [Fig. 6 *C* and *D*; (23)]. A recent synthesis of monthly resolved coral and bivalve records also confirms this pattern, estimating a reduction in ENSO variance of ~50% in the eastern Pacific and ~80% in the central Pacific during the interval between 6 and 3 ka (38).

Climate modeling studies reinforce this consensus. Mid-Holocene simulations consistently show weakened ENSO activity across a wide range of coupled climate models (39). Barr et al. (27) found a maximum in Walker circulation strength and a minimum in Niño 3.4 (equatorial east-west) sea surface temperature variance at 5 ka, pointing to a strengthened La Niña–like state (Fig. 6*G*). Tiwari et al. (40) highlight the role of vegetation–climate feedbacks, showing that “greening” of the Sahara during the African Humid Period intensified the Walker circulation via altered zonal gradients, thereby promoting La Niña-like conditions in the equatorial Pacific in the early Holocene.

Hydroclimate records further corroborate this picture. Eastern Pacific sites (Galápagos, Peru, Colombia) record low rainfall and dry conditions between ~8 and 5 ka [Fig. 6*H*; (28, 41, 42)], while western Pacific sites show opposing precipitation anomalies, consistent with a zonally asymmetric La Niña-like state [Fig. 6*I*; (27)]. Galápagos crater lakes show gypsum deposition during extended dry phases, with wet El Niño events generally less frequent between 6 and 4 ka (43).

Marine ecological data add a biological dimension to these physical climate metrics. Phytoplankton community structure in the East Equatorial Pacific shifted from coccolith- to diatom-dominated assemblages in the early Holocene, consistent with intensified upwelling and elevated primary productivity under persistent La Niña-like conditions (44, 45).

Together, the strength of evidence suggests that the cold-water coral hiatus we find at ~5 to 3.5 ka coincides with a unique low in ENSO variability over the last 25,000 y and a sustained La Niña-like state with significant implications for tropical climate, global teleconnections, and marine ecosystems.

### Oxygen Depletion and Loss of Cold-Water Coral Habitat at 5 ka.

Although surface-water primary production and the food it supplies to the deep has been identified as a key driver of cold-water coral habitat elsewhere [e.g., N. Atlantic; (8)], the habitat loss we find at ~5 ka in the East Equatorial Pacific was unlikely due to food shortage. A shift to a persistent La Niña-like state would have enhanced upwelling, stimulating productivity, thereby increasing the downward flux of organic matter (44). However, remineralization of this organic carbon would also lower pH and consume oxygen in subsurface waters, thus intensifying the oxygen minimum zone (17) (Fig. 3*B*). Subfossil preservation of older (>5 ka) corals argues against a significant reduction in pH or saturation state as the primary cause of coral habitat loss. This leaves oxygen depletion in excess of coral tolerance limits in the depth range of 200 to 1,000 m as the more plausible driver (e.g., refs. 7, 14, and 15). To evaluate this hypothesis, we examine proxy records of oxygen change across the last deglaciation and Holocene.

Sediment core records provide evidence that the deep Pacific (>2,000 m) was oxygen-depleted during the last glacial maximum and deglaciation facilitating storage of respired carbon. Redox-sensitive uranium concentrations and δ^13^C measurements in benthic foraminifera from equatorial Pacific cores suggest a substantial expansion of low-oxygen waters and a deeper respired carbon pool during the glacial period (46, 47). Similarly, benthic foraminiferal B/Ca, U/Ca, and δ^13^C records point to reduced carbonate ion concentrations and oxygen levels at ~2,000 m depth during the last glacial maximum, consistent with enhanced deep carbon storage (48). Excess-^230^Th and uranium records also indicate suboxic deep-water conditions in the East Equatorial Pacific during the glacial interval, followed by gradual deep-ocean oxygenation during deglaciation, concurrent with rising atmospheric CO_2_ (49).

While these deep-water records highlight the role of the Pacific in glacial carbon storage, they do not directly capture the oxygen conditions relevant to cold-water coral habitat in the upper 1,000 m. Global data syntheses indicate that the upper water column remained relatively oxygenated until the early Holocene, when a modern-style oxygen minimum zone shoaled to ~500 m (50, 51). Principal component analysis of deglacial sediment records (50) shows that oxygenation improved in the deep Pacific after the glacial, while an increasingly intense oxygen minimum zone began to develop in mid-waters. Stable isotope reconstructions from planktic foraminifera also support this changing oxygen content of the East Pacific—revealing an intense oxygen minimum zone during the Holocene, while deeper waters became more oxic (52).

Taken together, these studies suggest a fundamental vertical reorganization of the Pacific oxygen budget across the deglaciation: oxygen-depleted waters that had been confined to the abyss shifted upward into intermediate depths. For cold-water coral habitat in the East Equatorial Pacific, this transition would have been critical. During the last glacial and deglaciation, the deep Pacific was oxygen-poor while the upper 1,000 m remained well-oxygenated— conditions that were clearly favorable for cold-water corals as evidenced by high cold-water coral abundance, particularly during the Younger Dryas (Fig. 5*C*). By the early to mid-Holocene, however, the deep ocean became more oxygenated while the oxygen minimum zone at ~500 m formed, directly overlapping with coral habitat and producing persistently hypoxic conditions.

Modern East Equatorial Pacific corals are clearly well adapted to “extreme” conditions compared to other global habitats, tolerating oxygen levels far below the limits of other cold-water corals such as *D. pertusum* in the North Atlantic (12) and pH values as low as 7.6 (20, 21) (Fig. 2). Their disappearance at ~5 ka therefore implies conditions even more severe than today—either with oxygen depletion well below these thresholds or reduced seasonal variability that eliminated brief oxygenated intervals critical for growth and juvenile recruitment.

### Depth-Dependent Coral Habitat Responses to ENSO Variability.

Comparison with shallow-water coral records from the eastern tropical Pacific reveals that coral habitat loss was offset in timing but partially did temporally overlap across depth zones. Cold-water (deeper) corals in the Galápagos disappeared earlier, between ~5.0 and 3.5 ka, whereas shallow-water coral habitats declined later, from ~4.1 to 1.6 ka (29, 30, 53) (Fig. 6*J*). This pattern indicates that coral ecosystems at different depths responded differently to the evolving ENSO mean state and variability during the mid-Holocene.

Radiocarbon analysis in shallow-water corals from the Pacific coast of Panama show that the onset of shallow reef decline coincided with increased upwelling between ~4.5 and 3.8 ka, consistent with the establishment of persistent La Niña-like conditions (53). These findings indicate that La Niña–associated cooling and nutrient enrichment may have initiated stress in shallow-water coral ecosystems, similar to the timing at which subsurface oxygen depletion likely exceeded tolerance thresholds for cold-water corals.

Over longer timescales, however, shallow-water corals appear particularly sensitive to increases in ENSO variability and episodic El Niño warming. A synthesis of tropical Pacific coral growth and bleaching records shows that coral stress generally increases with higher long-term mean temperatures and enhanced El Niño variability (54). In this context, the return of stronger ENSO variability and frequent El Niño warming of surface waters after ~3 ka may have delayed the recovery of shallow-water coral habitats following the initial upwelling-driven decline (53).

Importantly, modern observations demonstrate that La Niña conditions are not universally benign for shallow reefs. Particularly cold sea-surface temperature anomalies during La Niña in recent decades have been linked to bleaching and mortality in Equatorial Pacific shallow-water corals (55, 56), highlighting that sustained cooling can also exceed coral thermal tolerances. Together, these observations underscore that both persistent La Niña-like states and periods of strong El Niño can negatively impact shallow-water corals, albeit through different mechanisms.

Disentangling the precise environmental controls on coral habitat loss in both deep- and shallow- water habitats remains challenging because the skeletal material required for coral-based geochemical reconstructions is, by definition, absent during coral hiatuses. As a result, interpretations must rely on integration of regional proxy records, climate model simulations, and modern analogues rather than direct coral archives during these periods.

### Impact of Future ENSO Change on Coral Habitat.

Projections of future ENSO behavior remain uncertain, with combined analyses of the Niño 3.4 index and CMIP models offering no clear consensus on whether variability will increase or decrease (57). Recent observations, however, indicate that multiyear La Niña events have become more frequent in recent decades (58). While these phases may influence thermal stress on shallow-water corals by cooling surface waters, their broader impacts may carry additional hidden risks for vulnerable deep-water ecosystems. Enhanced upwelling during La Niña in the East Equatorial Pacific likely intensifies oxygen depletion at mid-depths, a process that we link to the ~5 ka hiatus in cold-water coral occurrence. This sensitivity underscores the need to evaluate coral vulnerability across the full water column as ENSO evolves under anthropogenic climate change.

ENSO’s global teleconnections amplify these risks to habitat. The ~5 ka cold-water coral hiatus in the East Equatorial Pacific coincides with a mid-Holocene ENSO variability minimum and mirrors a similar interruption in coral records from the East Equatorial Atlantic [Carter and Knipovich Seamounts; 21 to 26°W; Fig. 5*G*; (14, 24)]. In the Atlantic, this gap, which occurred a few thousand years earlier in the early Holocene, has likewise been attributed to reduced mid-depth oxygen concentrations (14). Although the precise timing of coral collapse varies, the close temporal alignment between these events suggests that suppressed ENSO variability during the early to mid-Holocene may have disrupted tropical climate, upwelling, and ocean circulation in multiple basins. These changes highlight the far-reaching ecological consequences of a switch to a permanent La Niña–like state on cold-water coral habitats beyond the Pacific.

## Summary

Radiometric dating of corals from the submarine flanks of the Galápagos Islands provides the first evidence for a thriving cold-water coral ecosystem in this region extending back at least 117,000 y. These corals persisted through major episodes of global climate instability, including the last deglaciation, Heinrich events, and the Younger Dryas. The only significant disruption to this long record of persistence occurred at ~5 ka, lasting ~1.5 ky. This tropical climate event is not expressed in high latitude ice core records but coincides with a mid-Holocene reduction in ENSO variability and the development of more permanent La Niña–like conditions.

Comparison with oxygen-sensitive proxy records indicates that this habitat loss was most likely driven by a reorganization of oxygen content in the equatorial Pacific and the establishment of a particularly intense oxygen minimum zone at intermediate depths. This environmental stressor appears unique in the context of the last glacial cycle and highlights the sensitivity of cold-water corals to changes in oxygen availability.

Importantly, our findings reveal a depth-dependent and temporally offset response of coral habitats to tropical climate variability in the East Pacific. Cold-water corals were most vulnerable during the establishment of persistent La Niña-like conditions in the mid-Holocene, when enhanced upwelling and subsequent increased primary production intensified oxygen depletion at intermediate depths (200 to 1,000 m). Shallow-water coral habitats declined later, and their response reflects sensitivity both to sustained cooling and upwelling under La Niña–like states, as well as to increased ENSO variability and episodic El Niño warming (29). Together, these patterns show that ocean–climate states that may impact thermal stress for shallow-reef settings can simultaneously exacerbate subsurface hypoxia and compromise deep-sea coral ecosystems.

Recognizing the “unseen” nature of cold-water coral habitats is critical. Their ecological role as keystone taxa, yet vulnerability to climate-driven oxygen changes remain hidden from surface observations. Understanding both the longevity of this ecosystem and the environmental thresholds that have previously driven habitat loss provides valuable context for ongoing conservation efforts. These insights will be crucial for management strategies led by the Galápagos National Park, the Charles Darwin Research Foundation and Ecuadorian Institute for Oceanographic and Antarctic studies (INOCAR) as they work to safeguard marine biodiversity within this iconic Marine Protected Area.

### Cold-Water Coral Dating Methods.

#### Coral collection.

Coral samples (n = 787) were collected by human-operated submersible *Alvin* and ROV *SuBastian* during respective R/V *Atlantis* AT50-09 and R/V *Falkor* FKt230918 expeditions to the Galápagos in 2023 (Fig. 1). Subfossil scleractinian coral skeletons including colonial (*Dendrophyllia*, *Madrepora*) and solitary cup coral (*Caryophyllia*, *Desmophyllum*) genera, were recovered from 190 to 950 m depth (Fig. 1). Few dives targeted shallow waters (<300 m) as coral numbers were observed to be greatly reduced, with no fossil cold-water corals being recovered on dives AT50-09 5164 (~250 m) and FKt230918 dives 590, 599, 600, 601 (120 to 250 m) (20, 21). Results in this study are therefore unlikely a result of sampling bias toward deeper waters. Similarly, although many deeper dives were conducted for the collection of volcanic samples on these expeditions (down to >3,000 m), no subfossil scleractinian corals were found below ~1,000 m (20, 21).

An additional 151 corals were obtained from expeditions R/V *Melville* MV1007 (2010) and R/V *Nautilus* NA064 (2015). Of these, we present 123 new laser ablation reconnaissance dates, while 28 have published high-precision U-Th solution ages (18). Analytical methods and age uncertainties for both approaches are described below.

#### Rapid laser ablation reconnaissance U-series dating.

All corals were initially dated using laser ablation U-Th techniques, enabling rapid analysis of large numbers of specimens with typical uncertainties of ~7% (or ±1,000 y) for deglacial samples (59). Well-preserved skeletal fragments of solitary and colonial corals were cut to remove altered surfaces (e.g., recrystallized chalky material or endolithic borings), polished, and mounted onto stubs for ablation using a Photon Machines Analyte G2 193 nm laser. Isotopes ^230^Th (central ion counter) and ^238^U (Faraday cup) were measured simultaneously on a Neptune Multi-Collector Inductivity Coupled Plasma Mass Spectrometer (MC-ICP-MS) at the University of Bristol using established methods (59). Ages were calculated from measured ^230^Th/^238^U ratios, assuming that the initial ^234^U/^238^U of the sample is similar to modern seawater and negligible initial ^230^Th (59). Calculations used Newton-Raphson iteration, with ^230^Th/^238^U ratios corrected for background (laser cell gas blank) and scaled to an inorganic aragonite standard [VS001/1-A; 105.6 ± 1.9 ka; (59)]. U-Th dates measured by laser ablation agree well with precise isotope dilution results in corals where both techniques were applied (i.e., deglacial sample dates were generally within 10% of the high precision date; *SI Appendix*, Fig. S3). Where these more precise dates are available (see methods below) the isotope dilution U-Th date supersedes the laser ablation date in figures and results analysis.

#### Precise isotope dilution U-series dating.

Where sufficient well-preserved material permitted, selected samples (<35 ka) were more precisely dated using U-Th isotope-dilution techniques (24, 60), typically yielding ±100 y uncertainties (2SD) for deglacial ages. Corals were cut, chemically cleaned (oxidative and reductive), dissolved, and spiked with a well-calibrated (±4‰) ^236^U-^229^Th spike. U and Th were coprecipitated with iron hydroxides, isolated via anion-exchange columns, and analyzed by MC-ICP-MS at Bristol using sample-standard bracketing (U112a for U; SGS for Th). Measurements of standards HU1 and ThB yielded accuracy and internal precision better than ±1‰ (^234^U/^238^U) and ±2‰ for (^229^Th/^230^Th). ^229^Th and ^230^Th isotopes were measured via peak jumping on a secondary electron multiplier, with a pure ^236^U spike added to the Th fraction for normalization.

Ages (calendar B.P.; Year 1950) were calculated by Monte Carlo simulation (100,000 iterations) that incorporate analytical uncertainty, procedural blanks, and initial ^230^Th (24). We assume an initial atomic activity ratio of ^230^Th/^232^Th of 91.4 (18). To ensure quality control of coral dates and that skeletal material had remained in a closed system, 11 samples with high ^232^Th (>1,000 pg/g) were rejected. A further three corals with ages younger than 18 ka were also rejected for having initial δ^234^U ratios outside of the 3‰ range of the modern ocean [145.6‰; (61)]. None of the rejected corals were younger than 9 ka therefore the decision to exclude these corals does not affect study conclusions or the notable gap in coral occurrence beginning at 5 ka.

## Supplementary Material

Appendix 01 (PDF)

Dataset S01 (XLSX)

Movie S1. Spatial and temporal occurrence of cold-water corals around the Galápagos Islands study site in the East Equatorial Pacific since 22 ka. The size of the purple circles denotes the number of sub-fossil corals found at study site locations (open black circles; rounded to the nearest 0.1° of latitude and longitude for grouping similar dive localities) for each 1000-year interval. Histogram insert corresponds to that in Figure 5 (Main text) and compares coral dates to Greenland ice core δ^18^O temperature (and ice volume) record (1). Heinrich Stadials (HS), the Younger Dryas and the 8.2 kyr event (8.2) are shown as vertical blue bars. Base map from ArcGIS Ocean Basemap.

## Data Availability

Previously published data were used for this work (18). All other data are included in the manuscript and/or supporting information.
