# Supplementary material for: A millennium of cold-water coral habitat loss in the East Pacific during low ENSO variability in the mid- to late Holocene
Source: Proc Natl Acad Sci U S A. 2026 Apr 20;123(18):e2532081123. doi: 10.1073/pnas.2532081123 (PMC13142978; doi:10.1073/pnas.2532081123)
Supplement: Supplementary file 1 — Appendix 01 (PDF) [file pnas.2532081123.sapp.pdf]

PNAS

Supporting Information for

A millennium of cold-water coral habitat loss in the East Pacific during low ENSO variability in the mid- to late Holocene

Joseph A. Stewart [a*], Laura F. Robinson [a,b], Michelle L. Taylor [c], Daniel J. Fornari [d], Katleen Robert [e], Stuart Banks [f], Tianyu Chen [g], Tao Li [h], James Kershaw [a], Shannon Hoy [i], Qian Liu [a,h], Jessica D Gordon [c], Maria Luiza de Carvalho Ferreira [a,j], Ana Samperiz [k,l], Yingchu Shen [a], Yun-Ju Sun [a,m], Maoyu Wang [g]

[a] School of Earth Sci. University of Bristol, Queens Road, Bristol, BS8 1RJ, UK
[b] Department of Environment and Geography, University of York, York, UK.
[c] School of Life Sciences, University of Essex, UK
[d] Department of Geology & Geophysics, Woods Hole Oceanographic Institution, MA, USA
[e] Fisheries and Marine Institute of Memorial University, NL, Canada
[f] Charles Darwin Foundation, Av. Charles Darwin s/n, Puerto Ayora, Galápagos, Ecuador
[g] School of Earth Sciences and Engineering, Nanjing University, Nanjing, China
[h] State Key Laboratory of Palaeobiology and Stratigraphy, Nanjing Institute of Geology and Palaeontology, Nanjing, China.
[i] Seirios Solutions, LLC, Brookfield, New Hampshire, USA
[j] Department of the Geophysical Sciences, University of Chicago, Chicago, Illinois 60637, USA
[k] Florida State University, 1800 E. Paul Dirac Dr., Tallahassee FL 32310-3706
[l] School of Earth and Environmental Sciences, Cardiff University, Cardiff, CF10 3AT, UK
[m] Earth & Environmental Science, University of St Andrews, KY16 9TS

Corresponding author: Joseph A. Stewart
Email: joseph.stewart@bristol.ac.uk

This PDF file includes:

Supplementary Figure 1
Supplementary Figure 2
Supplementary Figure 3
Supplementary Figure 4
Legend for Movie S1
Legend for Dataset S1
SI References

Other supporting materials for this manuscript include the following:

Movie S1
Dataset S1

Figures

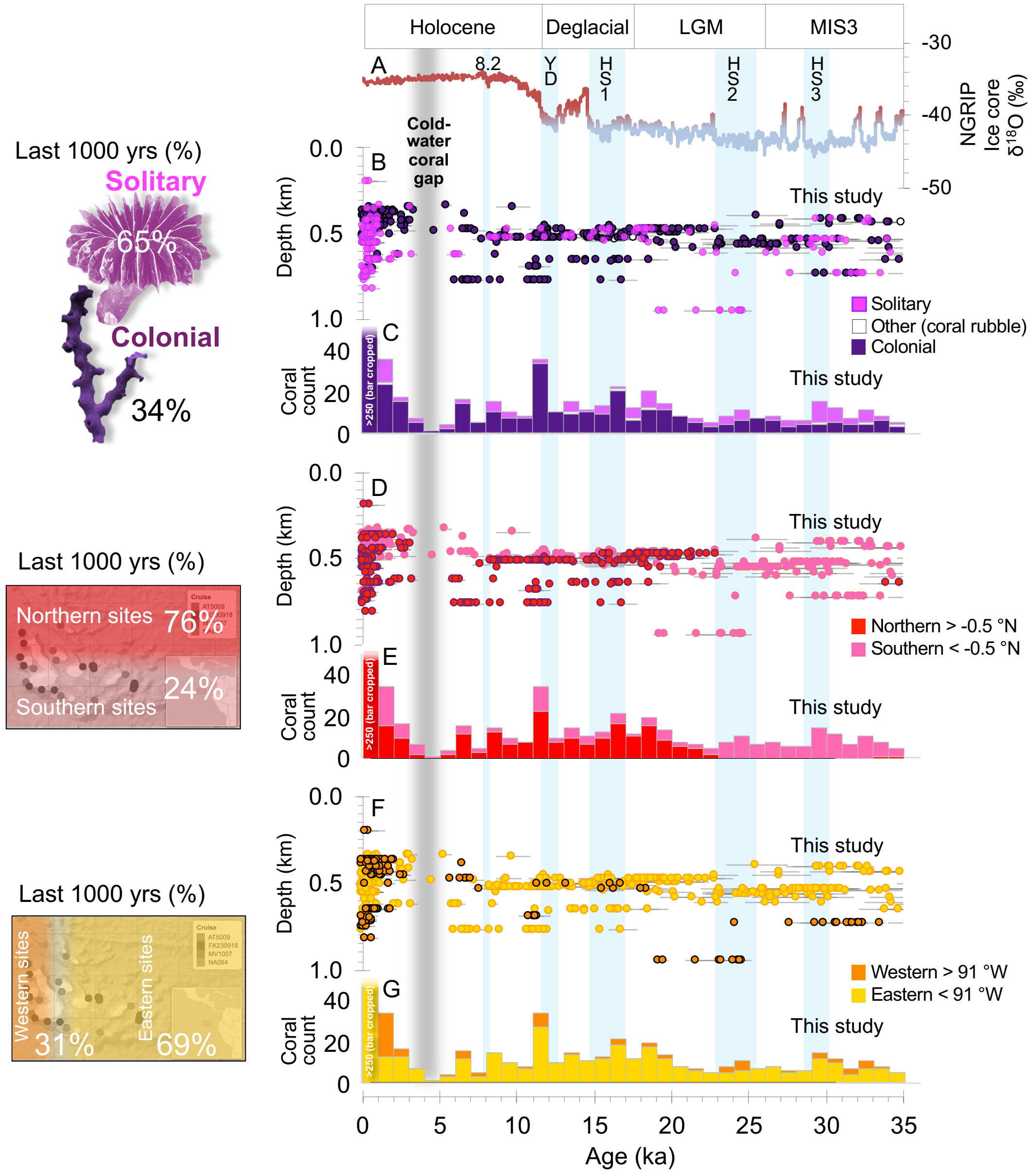

Supplementary Figure 1: East Equatorial Pacific scleractinian cold-water coral occurrence over the last 35 kyr divided by coral taxon and spatial distribution. (A) Greenland ice core $\delta^{18}O$ temperature (and ice volume) record (1). Depth distributions and histograms of sub-fossil scleractinian coral U-Th ages separated by: (B and C) Solitary and colonial corals; (D and E) North and south of -0.5 °N; (F and G) East and west of 91 °W (i.e. sites within the archipelago and open ocean facing sites)

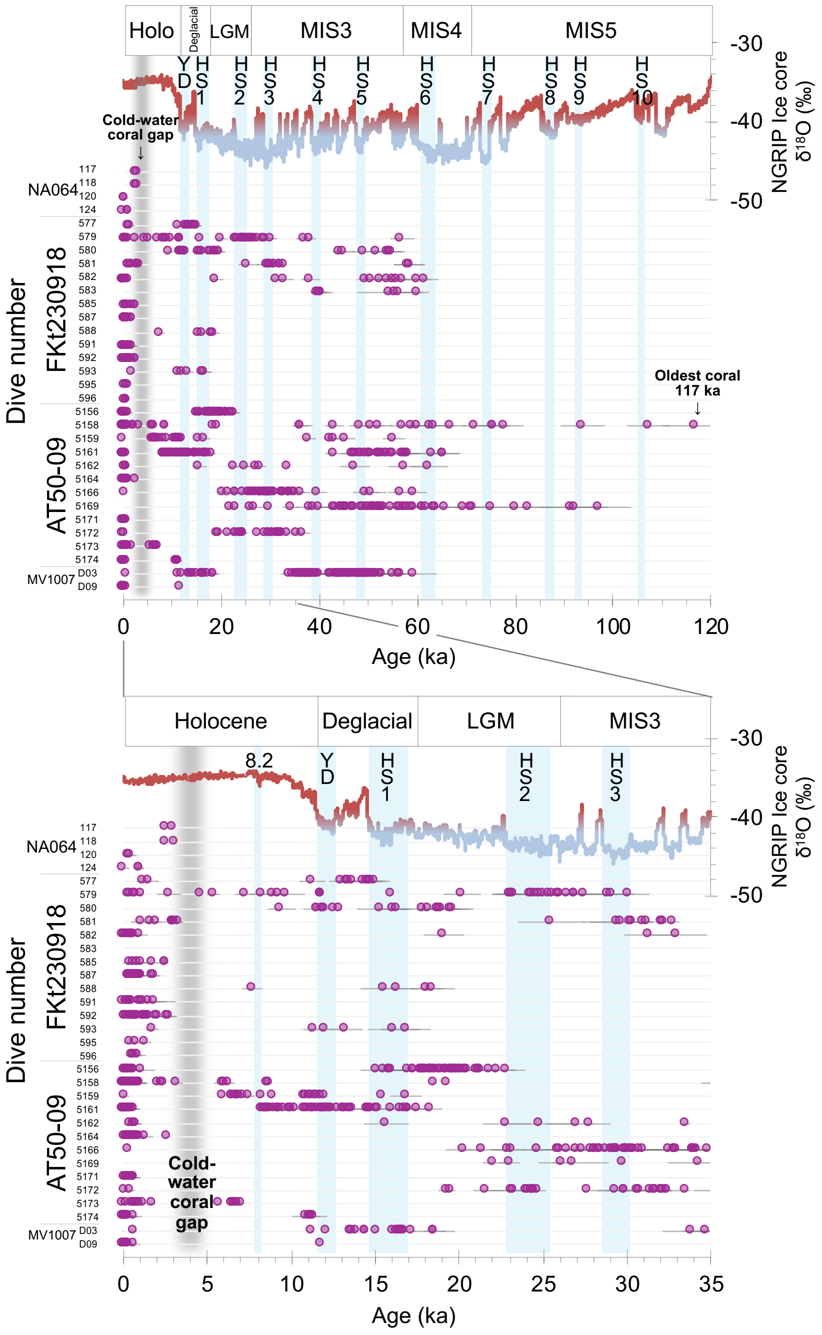

Supplementary Figure 2: East Equatorial Pacific scleractinian cold-water coral occurrence over the last 35 kyr separated by research cruise dive number. Note corals from the same dive (i.e. similar locations) yield corals occurring both before and after the cold-water coral gap (grey bar) suggesting that the hiatus is not due to sampling bias and each site only yielding corals of a single age.

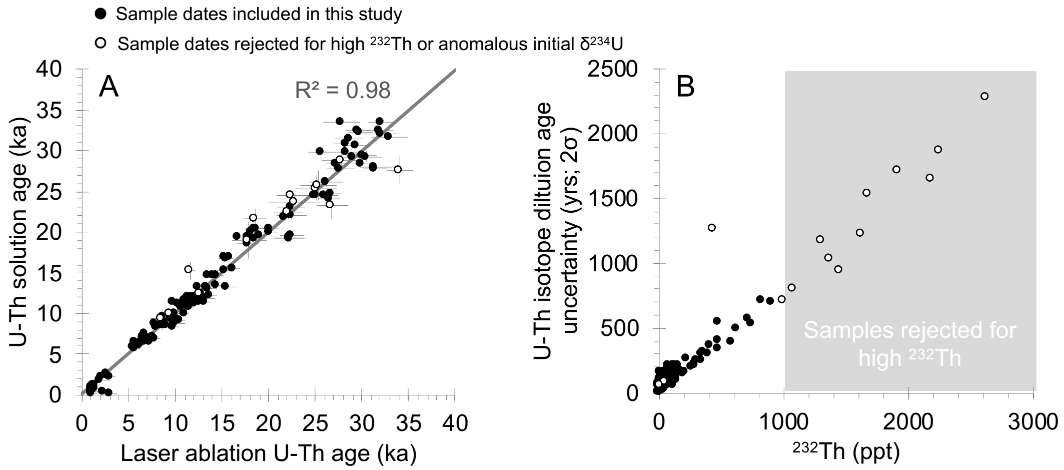

Supplementary Figure 3: Quality control of coral U-Th dates. (A) Comparison between low precision laser ablation and high precision isotope dilution U-Th dating of East Equatorial Pacific scleractinian cold-waters in this study. Error bars denote 2SD propagated uncertainty for each dating method. **(B)** Correlation between ^{232}Th and dating uncertainty detailing the removal of particularly high ^{232}Th (>1000 pg/g) due to their inflated age error. Samples rejected due to high ^{232}Th (>1000 pg/g) or initial δ^{234}U outside of the ± 3‰ range of modern seawater are shown as white circles. U-Th isotope dilution dates included for discussion are shown in black.

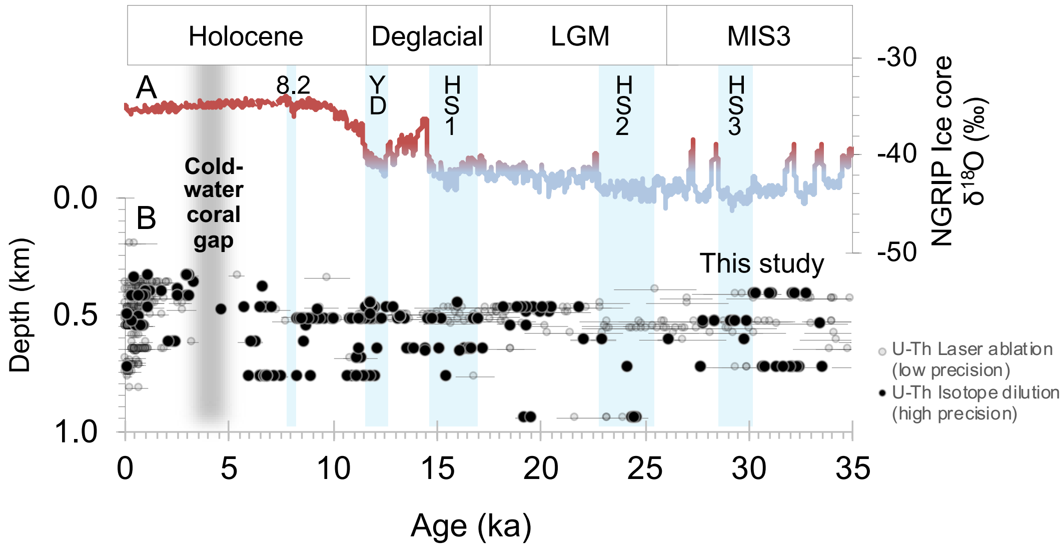

Supplementary Figure 4: East Equatorial Pacific scleractinian cold-water coral occurrence over the last 35 kyr – High precision U-Th isotope dilution dates are highlighted as darker black circles in panel B. Greenland ice core δ^{18}O temperature (and ice volume) record (1) shown in panel A.

Movie S1. Spatial and temporal occurrence of cold-water corals around the Galápagos Islands study site in the East Equatorial Pacific since 22 ka. The size of the purple circles denotes the number of sub-fossil corals found at study site locations (open black circles; rounded to the nearest 0.1° of latitude and longitude for grouping similar dive localities) for each 1000-year interval. Histogram insert corresponds to that in Figure 5 (Main text) and compares coral dates to Greenland ice core $\delta^{18}O$ temperature (and ice volume) record (1). Heinrich Stadials (HS), the Younger Dryas and the 8.2 kyr event (8.2) are shown as vertical blue bars. Base map from ArcGIS Ocean Basemap.

Dataset S1. Laser ablation and isotope dilution U-Th radiometric dates of cold-water coral samples collected on research expeditions AT50-09, FKt230918, MV1007, and NA064.

SI References

1. K. K. Andersen *et al.*, High-resolution record of Northern Hemisphere climate extending into the last interglacial period. *Nature* **431**, 147-151 (2004).
